# Antiviral Effect of Propylene Glycol against Envelope Viruses in Spray and Volatilized Forms

**DOI:** 10.3390/v15071421

**Published:** 2023-06-23

**Authors:** Yui Hirama, Shintaro Onishi, Ryunosuke Shibata, Hirohiko Ishida, Takuya Mori, Noriyasu Ota

**Affiliations:** 1 Biological Science Research, Kao Corporation, 2606 Akabane, Ichikai-machi, Haga-gun, Tokyo 321-3497, Japan; 2 Sensory Science Research, Kao Corporation, 2-1-3 Bunka, Sumida-ku, Tokyo 131-8501, Japan

**Keywords:** aerosol, antiviral, COVID-19, infectious diseases, influenza virus, propylene glycol

## Abstract

Severe acute respiratory syndrome coronavirus 2 (SARS-CoV-2) is highly contagious and continues to spread worldwide. To avoid the spread of infection, it is important to control its transmission routes. However, as methods to prevent airborne infections are lacking, people are forced to take measures such as keeping distance from others or wearing masks. Here, we evaluate the antiviral activity of propylene glycol (PG), which is safe, odorless, and volatile. PG showed pronounced antiviral activity against the influenza virus (IAV) at concentrations above 55% in the liquid phase. Given its IAV inactivation mechanism, which involves increasing the fluidity of the viral membrane, PG is expected to have a broad effect on enveloped viruses. PG showed antiviral activity against SARS-CoV-2. We also developed a system to evaluate the antiviral effect of PG in spray and volatilized forms. PG was found to be effective against aerosol IAV in both forms; the effective PG concentration against IAV in the vapor phase was 87 ppmv (0.27 mg/L). These results demonstrate that PG is an effective means for viral inactivation in various situations for infection control. This technology is expected to control the spread of current and future infectious diseases capable of causing outbreaks and pandemics.

## 1. Introduction

COVID-19 is an emerging infectious disease that was identified in late 2019. This disease is caused by the severe acute respiratory syndrome coronavirus 2 (SARS-CoV-2), a newly emerged single-stranded RNA virus of the Coronaviridae family [1,2]. Despite the serious countermeasures taken to control the spread of COVID-19 worldwide, mutant strains continue to appear and spread. In addition, other existing viruses, such as influenza, threaten to cause seasonal epidemics or global pandemics that occur every few years.

There are several routes of transmission of respiratory viruses from infected to non-infected persons, including indirect contact and the direct spraying of large droplets and droplet nuclei, which are extremely small, dry droplets that can remain suspended in the air for extended periods. In 2021, the World Health Organization and the US Centers for Disease Control and Prevention officially acknowledged that the inhalation of virus-laden aerosols is the main transmission mode for spreading COVID-19 in both short and long ranges [3].

It is important to control these infection routes to prevent viral infections. Sprays, disinfectants, and hand soaps, which can remove attached viruses, are effective in preventing indirect contact infections. In contrast, there are still few options for preventing droplet and airborne infections. Thus, preventive actions, such as wearing masks, increasing ventilation, and keeping a distance from others, are being taken. However, there are many situations in which these preventive actions cannot be followed. For example, masks must be removed when eating. Therefore, new technologies to inactivate viruses in the airspace are required.

Here, we focused on propylene glycol (PG) as a material for inactivating viruses in the airspace. PG has the advantage of being easy to apply in living spaces because it has no odor and a low safety risk [4,5]; it is even used as a food additive and in e-cigarette liquid solutions. In addition, the vapor pressure of PG is 14.8 Pa at 25 °C [6], making it as volatile as common fragrance materials that can be easily volatilized throughout a space. PG has shown antibacterial and antifungal activities against *Candida albicans, Staphylococcus aureus, Staphylococcus epidermidis, Streptococcus pyogenes A, Streptococcus mitis, Streptococcus mutans*, *E. coli,* Pneumococcus Type I, and *Staphylococcus albus* [7,8,9]. It has also been reported to inactivate influenza viruses via volatilized PG [10]. However, the mechanism of virus inactivation by PG and its antiviral effect on SARS-CoV-2 remain unknown. In this study, we elucidated the virus inactivation capability and mechanism of PG. Moreover, we verified the efficacy of PG as a virus inactivator in multiple forms, including liquid, volatilized, and spray forms, to prevent viral transmission. These results demonstrate that PG has the potential to be used as an antiviral agent against the infectious diseases currently spreading worldwide.

## 2. Materials and Methods

### 2.1. Chemicals and Reagents

Propylene glycol (PG), ethanol (99.5), distilled water, methanol, PBS, sodium acetate, sodium carbonate, and zanamivir were obtained from Fujifilm Wako Pure Chemical Corporation (Osaka, Japan). Mineral oil was purchased from Sigma-Aldrich Co. LLC (St. Louis, MO, USA).

### 2.2. Cells and Viral Culture

MDCK cells (CCL-34) were obtained from the American Type Culture Collection (Manassas, VA, USA) and maintained in minimum essential medium (Sigma-Aldrich Co. LLC) supplemented with 5% (*v/v*) heat-inactivated fetal bovine serum (Biosera, Kansas, MO, USA) and 50 μg/mL gentamicin sulfate solution (Fujifilm Wako Pure Chemical Corporation). Influenza A virus (IAV) strain A/Puerto Rico/8/1934 (H1N1; VR-1469) was obtained from the American Type Culture Collection. IAV was propagated at 37 °C using MDCK cells in serum-free medium (SFM; Thermo Fisher Scientific Japan K.K., Kanagawa, Japan) supplemented with acetylated trypsin (2 μg/mL) (Sigma-Aldrich Co. LLC) and gentamicin sulfate solution (50 μg/mL). The virus pellets were obtained according to some previous reports with minor modifications [11,12,13]. Briefly, the culture medium containing the propagated virus was centrifuged at 300× *g* for 5 min, the supernatant was then centrifuged at 13,000× *g* for 2 h, and the supernatant after centrifugation was discarded; the pellet was then re-suspended, and the solution was collected as a condensed viral solution.

### 2.3. Antiviral Activity in the Liquid Phase (IAV)

Antiviral activity evaluations were conducted following a standardized protocol in ASTM-E1052-20, with minor modifications. In brief, IAV solution (5 μL; 6.0 × 10^5^ FFU) and 45 μL of the evaluation sample were mixed and allowed to react for 5 min at 22–23 °C, and 950 μL of SFM was added to stop the reaction. PG and ethanol were used as the evaluation sample, and were diluted with distilled water to 10–90% (*v/v*)to 20–90% (*w/w*), respectively. The sample was immediately titrated using the focus-forming assay described below.

### 2.4. Antiviral Activity in the Vapor Phase (1 L)

Three cotton balls (Yamatokojo Co., Ltd., Osaka, Japan) soaked in a total of 1 mL PG were placed at the bottom of a 1 L glass bottle (Tokyo Garasu Kikai Co., Ltd., Tokyo, Japan) and stirred at 500 rpm using a magnetic stirrer (As One Corporation, Osaka, Japan) at 22–23 °C for 30 min. IAV solution (1.5 μL; 8.3 × 10^5^ FFU) was dried on the lid of a cryo vial (Thermo Fisher Scientific Japan K.K.) for 30 min at 22–23 °C. This lid was attached to the inside of a 1 L bottle cap. The cap was then replaced on the 1 L bottle with the IAV lid attached, exposing the lid to PG vapor for 30 min at 22–23 °C. After exposure, the lid was removed from the cap. The IAV on the lid was dissolved in SFM and immediately titrated using the focus-forming assay described below.

### 2.5. Analysis of PG Concentration inside the 1 L Bottle

Three cotton balls soaked in a total of 1 mL PG were placed at the bottom of a 1 L glass bottle and stirred at 500 rpm using a magnetic stirrer at 22–23 °C for 30 min. A cap with a 3.5 mm diameter hole was used for analysis, and a gas-adsorbent Tenax TA tube (Gerstel K.K., Tokyo, Japan) attached to a PTFE tube (NICHIAS Corporation, Tokyo, Japan) was inserted through the hole to collect 30 mL of air in a 1 L bottle using a mini pump (Sibata Scientific Technology Ltd., Tokyo, Japan). The collected air was analyzed using a thermal desorption gas chromatography/mass spectrometry system with a DB-WAX gas chromatography column (60 m × 250 μm × 0.25 μm; Agilent Technologies Japan, Ltd., Tokyo, Japan). The GC oven (Agilent 8890 equipped with a 5977B MSD; Agilent Technologies Japan) temperature was programmed as follows: initial oven temperature 40 °C, held for 3 min, raised to 70 °C at 6 °C/min, and then raised to 240 °C at 4 °C/min. The thermal desorption unit (TDU2; Gerstel K.K.) was set to split mode, and its temperature was programmed as follows: initial temperature 15 °C, increased to 260 °C at 120 °C/min, and held for 5 min. The cooled injection system (CIS4; Gerstel K.K.) temperature was programmed as follows: initial temperature −70 °C, raised to 260 °C at 12 °C/s, and held for 30 min. For the determination of PG, a certain amount of methanol diluent was injected into the Tenax TA tube, and then analyzed via thermal desorption gas chromatography/mass spectrometry, as described above. A calibration curve was prepared based on the detected PG values.

### 2.6. Antiviral Activity of Aerosol Viruses

Water or 60 *v/v*% PG solution was sprayed from the top of a 36 L suction box (350 mm × 490 mm × 240 mm; As One Corporation) using an accumulative pressure sprayer (Koshin Ltd., Kyoto, Japan), which was replaced with a nozzle for fine mist (KBN8010N, H. Ikeuchi & Co., Ltd., Osaka, Japan) for 10 s. The IAV solution (1.2 × 10^8^ FFU/mL) was sprayed using a compressor nebulizer (Omron Corporation, Kyoto, Japan) for 10 s and allowed to settle for 10 s. According to the manufacturer’s product information, the mass median aerodynamic diameter of the floating solution was 3 μm, and the particle sizes of the virus solution were generally considered to be in an aerosol state. The aerosol virus floating in the box was collected in a total of 3 mL of SFM in a bubbler (Shibata Scientific Technology Ltd., Tokyo, Japan) using an aspirator (Showa Science Co., Ltd., Tokyo, Japan) for 3 min. The virus that settled in two 6 cm φ dishes (AGC Techno Glass Co., Ltd., Shizuoka, Japan), placed in advance on the bottom of the box, was collected in a total of 3 mL of SFM. The experiment was conducted in a room with a controlled temperature of 22–23 °C and humidity of 45–55%.

### 2.7. Quantification of IAV via Focus-Forming Assay

The virus titer was measured using a focus-forming assay, as described previously [11,12,13], with minor modifications. In brief, MDCK cells were infected with IAV by adding diluted viral solution to confluent MDCK cells in SFM in a 12-well plate. The cells were then incubated for 30 min. After infection, the cells were washed with D-PBS (Fujifilm Wako Pure Chemical Corporation) and incubated for 18 h at 37 °C in SFM (2 mL/well) supplemented with 1.2% cellulose (Ceolus RC-591S; Asahi Kasei Corporation, Tokyo, Japan), acetylated trypsin (2 μg/mL), and gentamicin (50 μg/mL). After incubation, the cells were fixed with chilled methanol (Fujifilm Wako Pure Chemical Corporation) and stained for viral nucleoproteins using mouse antinucleoprotein antibody (Abcam plc, Cambridge, UK) as the primary antibody and horseradish peroxidase-linked goat antimouse IgG + IgM antibodies as the secondary antibodies (Jackson ImmunoResearch, West Grove, PA, USA). The viral foci count was determined as described previously [14]. The virus concentration was calculated from the results of three independent experiments and is reported as the number of focus-forming units per milliliter (FFU/mL).

### 2.8. Antiviral Activity in Liquid Phase (SARS-CoV-2)

Antiviral activity against SARS-CoV-2 was investigated by the Chubu Food & Environmental Safety Center Co., Ltd. (Shizuoka, Japan). The antiviral activity evaluations were conducted following a standardized protocol in ASTM-E1052-20, with minor modifications. Briefly, 3 μL of SARS-CoV-2 JPN/Kanagawa/KUH003 and 27 μL of the evaluation sample were mixed and allowed to react for 5 min at 22–23 °C. Then, 270 μL of Dulbecco’s Modified Eagle’s Medium (Nacalai Tesque, Inc., Kyoto, Japan), which was supplemented with 2% heat-inactivated fetal bovine serum, 100 units/mL penicillin, 100 μg/mL streptomycin, and 1 mg/mL geneticin, was added to stop the reaction. VeroE6/TMPRSS2 (JCRB1819) cells were infected with SARS-CoV-2 by adding a diluted viral solution. Infectious titers were measured using the standard TCID_50_ Behrens–Kraber method.

### 2.9. Hemagglutination Assay

IAV solution (5 μL; 5.0 × 10^8^ FFU) and 45 μL of the sample solution were mixed and allowed to react for 5 min at 22–23 °C. Then, 950 μL of PBS was added to stop the reaction. Fifty microliters of this solution were diluted two-fold on a U-bottomed 96-well microtiter plate. After serial dilutions, 50 mL of 0.7% guinea pig whole blood cells was added to each well and mixed gently. The plate was left for 2 h at 4 °C, and HA units were measured.

### 2.10. Neuraminidase Assay

IAV solution (5 μL; 5.0 × 10^8^ FFU) and 45 μL of the sample solution were mixed and allowed to react for 5 min at 22–23 °C. Then, 950 μL PBS was added to stop the reaction. Fifty microliters of this solution were mixed with 25μL of 0.25 mM 4-methylumbelliferyl N-acetyl-a-D-neuraminic acid sodium salt (4MU–Neu5Ac; Carbosynth Ltd., Berkshire, UK) in 100 mM sodium acetate buffer (pH 6) and incubated at 37 °C for 30 min. The enzyme reaction for 4MU-Neu5Ac was stopped by adding 150 μL of 100 mM sodium carbonate buffer (pH 10.7). Fluorescence was quantified using a microplate reader (GloMax^®^ Discover; Promega, Madison, WI, USA) with excitation and emission wavelengths of 355 nm/460 nm for 4-MU. The relative NA inhibition value was calculated as 0% when only water was added and 100% when no IAV was added.

### 2.11. Laurdan Assay

Laurdan (6-dodecanoyl-2-dimethylamino naphthalene; Cayman Chemical Company, Ann Arbor, MI, USA) was dissolved in dimethyl sulfoxide (Sigma-Aldrich Co. LLC), and 20 mM stock solution was prepared and added to IAV (1 × 10^9^ FFU) to a final concentration of 20 μM. This mixture was incubated at 37 °C for 10 min and centrifuged at 21,600× *g* at 4 °C for 60 min. The supernatant was removed, and the pellet was suspended in 200 μL of PBS. IAV without laurdan was also prepared using the same method. The viral liquid (20 μL) and sample solution (180 μL) were mixed and allowed to react for 5 min on a black 96-well microtiter plate. Fluorescence was measured using a microplate reader with excitation and emission wavelengths of 440 nm/490 nm. After subtracting the fluorescence value of the sample without laurdan from the fluorescence value of the sample with laurdan added, the generalized polarization (GP) was calculated using the following formula:

GP = (I_440_ − I_490_)/(I_440_ + I_490_),

where I_440_ and I_490_ refer to the emission intensities at these wavelengths.

### 2.12. Statistical Analysis

The results from multiple experiments are expressed as mean ± S.E. All data were analyzed using IBM SPSS Statistics Version 24 (IBM Japan, Ltd., Tokyo, Japan). Student’s *t*-tests or one-way ANOVA followed by Dunnett’s test were used for statistical comparisons between groups. Statistical significance was set at *p* < 0.05.

## 3. Results

### 3.1. Concentration of PG That Exerts an Antiviral Effect and Its Mechanism in the Liquid Phase

**Figure 1:**
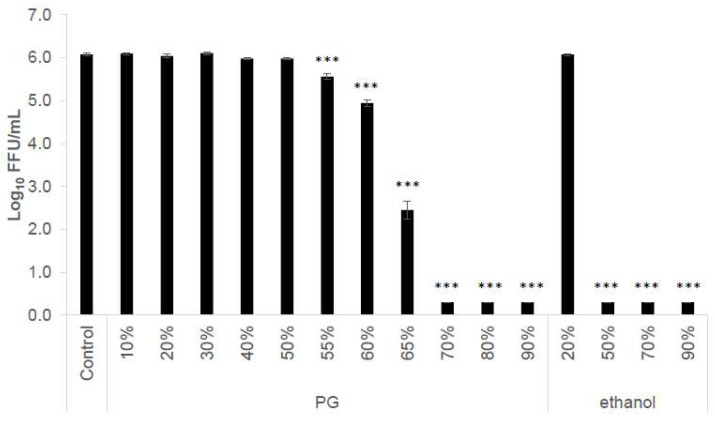
**Antiviral activity of serial dilutions of propylene glycol (PG) against influenza A virus (IAV) in the liquid phase.** IAV was mixed with different concentrations of PG (*v/v*%), ethanol (*w/w*%), or water (control) in a ratio of 1:9 for 5 min at 22–23 °C, and the remaining viral infectivity was measured. Results are presented as log_10_ FFU/mL and expressed as the mean ± S.E. of three independent experiments. The detection limit of the assay was 0.3 log_10_ FFU/mL. Statistical significance was calculated using one-way ANOVA followed by Dunnett’s test, compared to the control (*** *p* < 0.001).

The antiviral activities of different PG concentrations in the liquid phase against the IAV were examined. No activity was observed at PG concentrations < about 50%, but the activity increased remarkably to a 1.13 log_10_ reduction at a concentration of 60%, 3.62 log_10_ at 65%, and >5.77 log_10_ (the upper measurement limit) at ≥70% (Figure 1).

**Figure 2:**
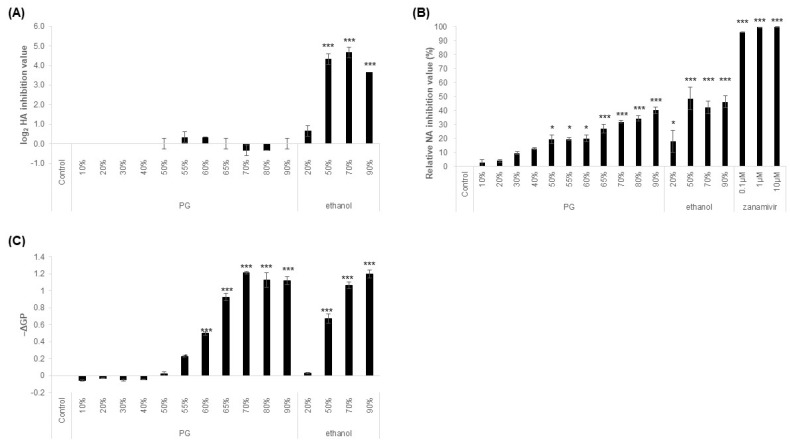
**Mechanism of antiviral activity of serial dilutions of propylene glycol (PG).** Influenza A virus (IAV) was mixed with different concentrations of PG (*v/v*%), ethanol (*w/w*%), zanamivir (μM), or water (control) in a ratio of 1:9 for 5 min at 22–23 °C. The results are expressed as the mean ± S.E. of three independent experiments. Statistical significance was calculated using one-way ANOVA followed by Dunnett’s test, compared to the control (* *p* < 0.05, *** *p* < 0.001). (**A**) HA inhibition activity of serial dilutions of PG and ethanol. The log_2_ HA inhibition value was calculated as zero for the control condition. (**B**) NA activity of serial dilutions of PG, ethanol, and zanamivir. The relative NA inhibition value was calculated as 100% when the control was added and 0% when no IAV was added. Zanamivir, a neuraminidase inhibitor, was used as a positive control. (**C**) Membrane fluidity of serial dilutions of PG and ethanol. The fluorescence was measured with excitation and emission wavelengths of 440 nm/490 nm, and the GP values were calculated. The −ΔGP value was calculated to be 0 for the control condition.

**Figure 3:**
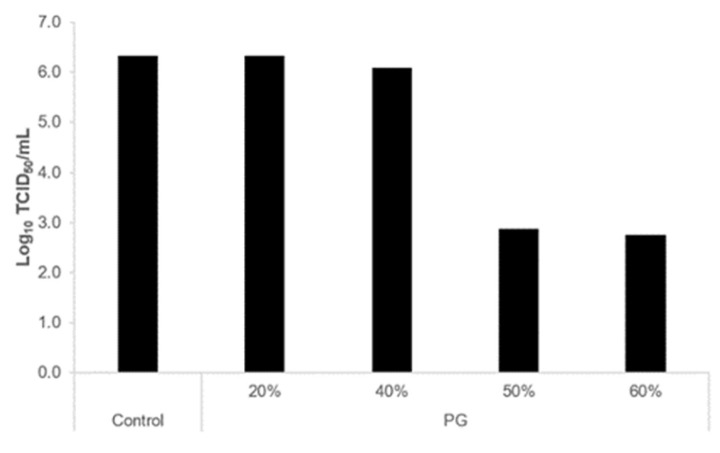
**Antiviral activity of serial dilutions of propylene glycol (PG) against SARS-CoV-2 in the liquid phase.** SARS-CoV-2 was mixed with different concentrations of PG (*v/v*%) or water (control) in a ratio of 1:9 for 5 min at 22–23 °C, and the remaining viral infectivity was measured. TCID_50_ was calculated twice using an independent assay, and the same results were obtained in both cases. The results are presented as log_10_ TCID_50_/mL. The detection limit was 2.76 log_10_ TCID_50_/mL.

To elucidate the virus inactivation mechanism of PG, we measured the inhibitory activity of hemagglutinin (HA) and neuraminidase (NA), which are important membrane proteins for IAV infection. PG had no effect on HA activity (Figure 2A). It inhibited NA in a concentration-dependent manner, but this effect was significantly weaker than that of zanamivir, a neuraminidase inhibitor (Figure 2B). Ethanol at concentrations >50% significantly inhibited HA (Figure 2A), but its inhibitory activity against NA was comparable to that of PG (Figure 2B). We then tested the effect of different concentrations of PG on lipid bilayers. Membrane fluidity was increased at PG concentrations >55% (Figure 2C). Furthermore, PG showed antiviral activity against another enveloped virus, SARS-CoV-2, at concentrations ≥50% (Figure 3).

### 3.2. Inactivation Effect of PG Mist on Aerosol Viruses

**Figure 4:**
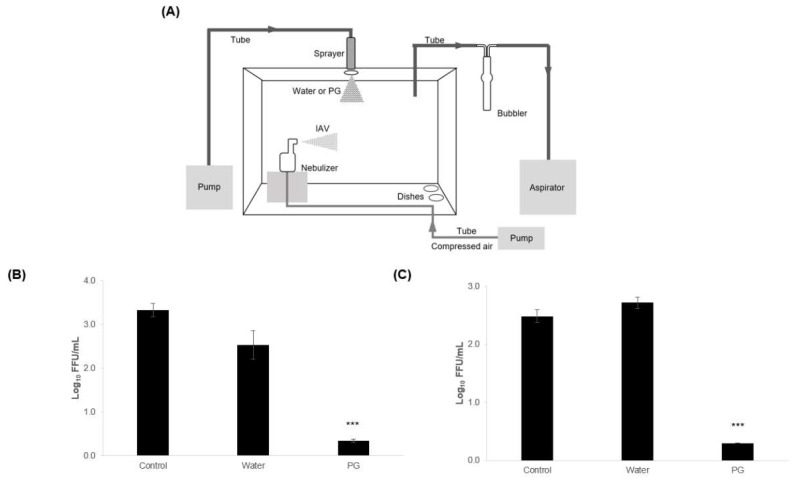
**Antiviral activity of propylene glycol (PG) mist against aerosolized influenza A virus (IAV).** (**A**) Diagram of the experimental system. Water or 60 *v/v*% PG was sprayed in a 36 L box using an accumulative pressure sprayer for 10 s and the virus solution was sprayed using a compressor nebulizer for 10 s at the same time. Nothing was sprayed in the control. The box was then allowed to settle for 10 s. The virus floating in the box was collected in a serum-free medium in a bubbler using an aspirator for 3 min. The virus that had fallen on two empty dishes placed in advance on the bottom of the box was collected in a serum-free medium. (**B**) Virus titer of floating virus in the 36 L box. (**C**) Virus titer of fallen virus in the dishes. Results are presented as log_10_ FFU/mL and expressed as means ± S.E. of four independent experiments. Statistical significance was calculated using one-way ANOVA, followed by Dunnett’s test, compared to the control (*** *p* < 0.001).

We examined the antiviral activity of PG mist by spraying it inside a 36 L experimental box (Figure 4A). The virus solution was spread as an aerosol, while water and a 60% PG solution were sprayed simultaneously using a pump. The number of viruses floating in the space and the number falling to the bottom of the box were measured. The results showed that, when spraying with 60% PG, the number of viruses suspended in the air was reduced by approximately 3 log_10_, and that of fallen viruses dropped by at least 2 log_10_ compared to the controls without mist (Figure 4B,C). Moreover, when only water was sprayed, although the number of viruses suspended in the air decreased by 0.8 log_10_, the number of fallen viruses increased slightly.

### 3.3. Antiviral Activity of PG in the Vapor Phase

**Figure 5:**
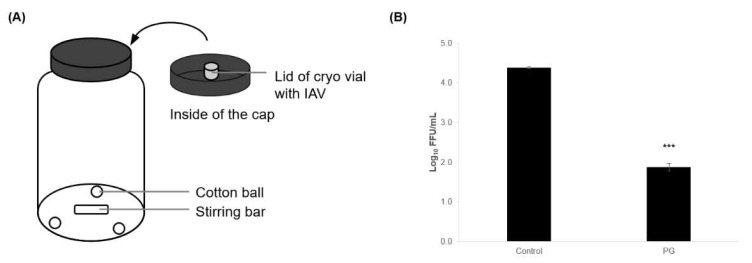
**Antiviral activity of propylene glycol (PG) against influenza A virus (IAV) in the vapor phase.** (**A**) Diagram of the experimental setting of 1 L glass bottle evaluation system. Three cotton balls soaked in PG or mineral oil (control) were placed at the bottom of the 1 L glass bottle. The lid containing IAV was attached to the cap of the 1 L bottle and exposed to PG vapor for 30 min. The remaining viral infectivity was measured. (**B**) Results are presented as log_10_ FFU/mL and expressed as means ± S.E. of three independent experiments. The statistical significance between each pair was calculated using Student’s *t*-test (*** *p* < 0.001).

We examined the antiviral activity of PG in the vapor phase. To confirm the effective concentration of volatilized PG, we constructed an evaluation system in a 1 L scale glass bottle and measured the antiviral activity and PG concentration in the space (Figure 5A). The results showed that volatilized PG in a 1 L space reduced influenza viruses by 2.5 log_10_ (Figure 5B), and the PG concentration under these conditions was 87 ppmv (0.27 mg/L, data not shown; measurement method is described in the Materials and Methods section).

## 4. Discussion

The goal of this study was to elucidate the virus inactivation capability and mechanism of PG, which is known as a virus inactivator, and to verify the efficacy of PG as a virus inactivator in multiple forms, including liquid, volatilized, and sprayed forms, to prevent viral transmission.

The functioning of the IAV surface protein HA was not affected by PG. Compared with zanamivir, the NA inhibitory effect of PG was lower and was considered to be insufficient in explaining its antiviral activity. However, PG elicited a remarkable increase in membrane fluidity, showing antiviral activity at concentrations > 55%. It is reported that antiviral agents targeting the envelope affect the fluidity of the viral lipid membrane and inhibit the fusion of viral and cell membranes, which is essential for viral infection [15,16]. Although the effects of PG on viral proteins other than HA and NA and viral RNA have not been evaluated, our results suggest that the change in the membrane ordering of the IAV envelope by PG contributes highly to the inactivation effect. It has been reported that 55% PG solution has the lowest excess molar enthalpy [17], and it is possible that changes in excess molar enthalpy affected its antiviral activity. Similarly, ethanol shows a rapid increase in antibacterial activity at concentrations higher than 30–40 *w/w*%, at which its excess molar enthalpy is the lowest [18]. In highly concentrated ethanol solutions, ethanol molecules form polymer-like structures via hydrogen bonds and hydrophobic bonds and aggregate with water molecules to form clusters with large hydrophobic surfaces [19]. They are more likely to interact with hydrophobic lipid bilayers in this form and are thus thought to have higher antibacterial and antiviral activities. It is possible that PG and water also form clusters, suggesting that the same phenomenon may occur in PG.

PG solution showed inactivation capability not only against the IAV but also against SARS-CoV-2 in this study. Even though the PG concentration was the same, the level of antiviral activity differed between IAV and SARS-CoV-2. This may be caused by differences in the composition of the lipid bilayer and the types of protein on the viral surface. It is highly likely that PG could also inactivate other enveloped viruses, although a further examination is needed. Moreover, it remains to be verified whether PG has an inactivating effect on non-enveloped viruses.

We constructed an experimental model of droplet and airborne transmission. In this evaluation, the number of viruses floating in the space was slightly reduced, but the number of fallen viruses with infectivity was slightly increased by water spraying. This suggests that spraying water droplets facilitates viruses in the space falling to the ground but does not inactivate them. In contrast, the reduction in viruses in the airspace by PG mist is thought to be largely due to the contribution of inactivation.

Several inactivator technologies that remove viruses in airspaces have been commercialized, including chlorine dioxide, air cleaners, and ultraviolet rays. However, some of these technologies have been confirmed to be effective only in small, enclosed spaces or on viruses attached to surfaces [20,21]. Their effectiveness in the actual airspaces has hardly been verified, making some of their effects unclear [22,23]. PG was known to have antiviral properties, but its practical application was difficult due to its high viscosity at high concentrations. However, now that the concentration of volatilized PG with a virus-inactivating effect has been revealed in this study, it will be easier to use lower concentrations of PG in spray form. To clarify whether PG can exert antiviral activity in a living space, it is necessary to examine the antiviral activity of PG in spaces that are larger than 36 L and to reproduce the ventilation conditions of real situations.

It is reported that PG vapor shows a high antiviral effect in the airspace for a short time and at low concentrations [24,25]. The volatilized PG evaluation on the 1 L scale in our study exhibited 2.5 log_10_ viral reduction at 87 ppmv (0.27 mg/L) of PG, as this antiviral activity is equivalent to approximately 60–65% of that in the liquid phase evaluation. Puck et al. and our study suggest that the antiviral effect is caused by the condensation of the PG vapor molecules on the virus-containing particles, so that an antiviral concentration of PG accumulates around the viruses [25]. In animal experiments, the death rate of influenza-virus-infected mice decreased by exposure to a concentration of 1 g of PG per 2,000,000 mL of air (0.5 mg/L) [10]. As the PG concentration falls below the saturation point, the rapidity of its bactericidal action against *Staphylococcus albus* decreases progressively, and 0.16 mg/L of PG was found to exert no killing effects, even at extremely low *Staphylococcus albus* concentrations [9]. In contrast, 30 min exposure to 0.05 mg/L of PG vapor was found to cause a reduction of 95% or more in the number of Pneumococcus Type I [9]. The differences in the effective concentrations may be owing to differences in the types of pathogens, number of pathogens, PG vaporization method, reaction time, temperature, and humidity between this study and previous reports. Since our study only demonstrated the effect of volatilized PG on IAV at a single concentration (0.27 mg/L), further studies validating several volatilized PG concentrations are needed to understand the effective concentration. Moreover, it will be necessary to determine the effective concentration in consideration of the condition of the living spaces and the target pathogens, as well as to confirm the safety of the concentration.

Several volatile compounds have been reported to exhibit antiviral activity, although only limited reports have evaluated such compounds in the vapor phase [26,27,28]. Aromatic volatile compounds and essential oils are known to have antiviral activity [29,30]. However, these compounds have limitations in terms of fragrance strength when sprayed in a space; their strong odor makes it difficult to use them in a space with people. In addition, inhalation toxicity becomes an issue when compounds are sprayed into a space. For example, octanal has been reported to have antiviral activity [27] but is known to display inhalation toxicity at high concentrations [31], limiting the concentration that can be used in a living space. In contrast, PG is commonly used as a solvent in various daily products and food additives, and its inhalation toxicity is extremely low [4,5,32]. The concentration of volatilized PG with an antiviral effect revealed in this study (0.27 mg/L) is lower than the No Observed Adverse Effect Level (1 mg/L) published by the European Chemicals Agency [33]. The use of volatile compounds is preferable for controlling airborne and contact transmission, and spraying PG into the airspace is one of the effective technologies with few safety concerns in human living spaces.

Our study has the following limitations. The virus inactivation effect of PG was only evaluated in a maximum space of 36 L. The temperature and humidity in the laboratory were controlled, but not in the box; the virus inactivation effect of PG may vary depending on different conditions, such as temperature, humidity, and space. In addition, the virus sprayed by nebulizer may exist unevenly in the box, but our experiment could only evaluate the antiviral effect of PG in the direction of spraying. In order to evaluate the inactivation effect in the entire space, it is necessary to improve the evaluation model, for example, by changing the virus spraying method. Also, the viruses we used in this study were prepared using cell culture, meaning they have different conditions to the airborne viruses present in the droplets and aerosols in terms of saliva components (protein amount), amount of water, salinity, and pH. These differences might affect the antiviral activity of PG. We will continue to study these limitations and hope that this technology can be used to control the infectious diseases spreading worldwide, such as COVID-19.

## Data Availability

All relevant data are included in the manuscript.

## References

[1] Pal M., Berhanu G., Desalegn C., Kandi V. (2020). Severe acute respiratory syndrome Coronavirus-2 (SARS-CoV-2): An update. Cureus.

[2] V’Kovski P., Kratzel A., Steiner S., Stalder H., Thiel V. (2021). Coronavirus biology and replication: Implications for SARS-CoV-2. Nat. Rev. Microbiol..

[3] Wang C.C., Prather K.A., Sznitman J., Jimenez J.L., Lakdawala S.S., Tufekci Z., Marr L.C. (2021). Airborne transmission of respiratory viruses. Science.

[4] Suber R.L., Deskin R., Nikiforov I., Fouillet X., Coggins C.R. (1989). Subchronic nose-only inhalation study of propylene glycol in Sprague-Dawley rats. Food Chem. Toxicol..

[5] Uddin M. (2018). A review on the safety of inhalation of propylene glycol in E-cigarettes. World J. Pharm. Pharm. Sci..

[6] US EPA (2021). Estimation Programs Interface Suite^TM^ for Microsoft^®^.

[7] Kinnunen T., Koskela M. (1991). Antibacterial and antifungal properties of propylene glycol, hexylene glycol, and 1,3-butylene glycol in vitro. Acta Derm. Venereol..

[8] Nalawade T.M., Bhat K., Sogi S.H. (2015). Bactericidal activity of propylene glycol, glycerine, polyethylene glycol 400, and polyethylene glycol 1000 against selected microorganisms. J. Int. Soc. Prev. Community Dent..

[9] Puck T.T., Robertson O.H., Lemon H.M. (1943). The bactericidal action of propylene glycol vapor on microorganisms suspended in air: II. The influence of various factors on the activity of the vapor. J. Exp. Med..

[10] Robertson O.H., Loosli C.G., Puck T.T., Bigg E., Miller B.F. (1941). The protection of mice AGAINST infection WITH air-borne influenza virus by means of propylene glycol vapor. Science.

[11] Saha R.K., Takahashi T., Suzuki T. (2009). Glucosyl hesperidin prevents influenza A virus replication in vitro by inhibition of viral sialidase. Biol. Pharm. Bull..

[12] Saha R.K., Takahashi T., Kurebayashi Y., Fukushima K., Minami A., Kinbara N., Ichitani M., Sagesaka Y.M., Suzuki T. (2010). Antiviral effect of strictinin on influenza virus replication. Antivir. Res..

[13] Onishi S., Mori T., Kanbara H., Habe T., Ota N., Kurebayashi Y., Suzuki T. (2020). Green tea catechins adsorbed on the murine pharyngeal mucosa reduce influenza A virus infection. J. Funct. Foods.

[14] Takahashi T., Murakami K., Nagakura M., Kishita H., Watanabe S., Honke K., Ogura K., Tai T., Kawasaki K., Miyamoto D. (2008). Sulfatide is required for efficient replication of influenza A virus. J. Virol..

[15] Song J.M., Seong B.L. (2010). Viral membranes: An emerging antiviral target for enveloped viruses?. Expert Rev. Anti Infect. Ther..

[16] Vigant F., Jung M., Lee B. (2010). Positive reinforcement for viruses. Chem. Biol..

[17] Nagamachi M.Y., Francesconi A.Z. (2006). Measurement and correlation of excess molar enthalpy for (1,2-propanediol, or 1,3-propanediol, or 1,4-butanediol+water) at the temperatures (298.15, 323.15, and 343.15) K. J. Chem. Thermodyn..

[18] Beppu M., Nakazima M., Katahira R. (1990). Differential scanning colorimetric thermography of water-alcohol mixtures and bactericidal action. J. Food Hyg. Soc. Jpn..

[19] Nishi N., Saita Y. (1994). Clusters in whiskey. Chem. Ind..

[20] Ogata N., Shibata T. (2008). Protective effect of low-concentration chlorine dioxide gas against influenza A virus infection. J. Gen. Virol..

[21] Kitagawa H., Nomura T., Nazmul T., Omori K., Shigemoto N., Sakaguchi T., Ohge H. (2021). Effectiveness of 222-nm ultraviolet light on disinfecting SARS-CoV-2 surface contamination. Am. J. Infect. Control.

[22] Nishimura H. (2016). Evaluation of the authenticity of practical usefulness of a commercial chemical product that claims the virucidal ability by releasing gas of chlorine dioxide: Investigation on inactivation of Air-borne influenza virus under a room temperature and humidity of the winter season. Jpn. J. Environ. Infect..

[23] Hammond A., Khalid T., Thornton H.V., Woodall C.A., Hay A.D. (2021). Should homes and workplaces purchase portable air filters to reduce the transmission of SARS-CoV-2 and other respiratory infections? A systematic review. PLoS ONE.

[24] Puck T.T. (1947). The mechanism of aerial disinfection by glycols and other chemical agents: II. An analysis of the factors governing the efficiency of chemical disinfection of the air. J. Exp. Med..

[25] Puck T.T. (1947). The mechanism of aerial disinfection by glycols and other chemical agents: I. Demonstration that the germicidal action occurs through the agency of the vapor phase. J. Exp. Med..

[26] Vimalanathan S., Hudson J. (2014). Anti-influenza virus activity of essential oils and vapors. Am. J. Essent. Oils Nat. Prod..

[27] Hayashi K., Kamiya M., Hayashi T. (1995). Virucidal effects of the steam distillate from Houttuynia cordata and its components on HSV-1, influenza virus, and HIV. Planta Med..

[28] Usachev E.V., Pyankov O.V., Usacheva O.V., Agranovski I.E. (2013). Antiviral activity of tea tree and eucalyptus oil aerosol and vapour. J. Aerosol Sci..

[29] Astani A., Reichling J., Schnitzler P. (2011). Screening for antiviral activities of isolated compounds from essential oils. Evid. Based Complement. Alternat. Med..

[30] Choi H.J. (2018). Chemical constituents of essential oils possessing anti-influenza A/WS/33 virus activity. Osong Public Health Res. Perspect..

[31] Song M.K., Lee H.S., Choi H.S., Shin C.Y., Kim Y.J., Park Y.K., Ryu J.C. (2014). Octanal-induced inflammatory responses in cells relevant for lung toxicity: Expression and release of cytokines in A549 human alveolar cells. Human Exp. Toxicol..

[32] Robertson O.H., Loosli C.G. (1947). Tests for the chronic toxicity of propylene glycol and triethylene glycol on monkeys and rats by vapor inhalation and oral administration. J. Pharmacol. Exp. Ther..

[33] Toxicological Summary of Propane-1,2-diol. https://echa.europa.eu/registration-dossier/-/registered-dossier/16001/7/1.

